# Guggulsterone Activates Adipocyte Beiging through Direct Effects on 3T3-L1 Adipocytes and Indirect Effects Mediated through RAW264.7 Macrophages

**DOI:** 10.3390/medicines6010022

**Published:** 2019-01-31

**Authors:** Colette N. Miller, Janaiya S. Samuels, Yusra Azhar, Ashish Parmar, Rangaiah Shashidharamurthy, Srujana Rayalam

**Affiliations:** 1 Department of Animal and Dairy Sciences, University of Georgia, Athens, GA 30605, USA; colettenmiller@gmail.com; 2 Department of Foods and Nutrition, University of Georgia, Athens, GA 30605, USA; 3 Department of Pharmaceutical Sciences, School of Pharmacy, Philadelphia College of Osteopathic Medicine-GA Campus, Suwanee, GA 30024, USA; jshanelle1990@gmail.com (J.S.S.); yusraaz@pcom.edu (Y.A.); ashishpa@pcom.edu (A.P.); rangaiahsh@pcom.edu (R.S.)

**Keywords:** guggulsterone, obesity, 3T3-L1, beige adipocytes, RAW264.7, M2 polarization

## Abstract

**Background:** Plant-derived phytochemicals have been of emerging interest as anti-obesity compounds due to their apparent effects on promoting reduced lipid accumulation in adipocytes. Despite such promising evidence, little is known about the potential mechanisms behind their anti-obesity effects. The aim of this study is to establish potential anti-obesity effects of the phytochemical guggulsterone (GS). **Methods:** Mature 3T3-L1 adipocytes were treated with GS, derived from the guggul plant native in northern India, to investigate its effects on mitochondrial biogenesis and adipocyte “beiging.” Further, to explore the relationship between macrophages and adipocytes, 3T3-L1s were treated with conditioned media from GS-treated RAW264.7 macrophages. Markers of mitochondrial biogenesis and beiging were measured by western blot. **Results:** GS treatment in adipocytes resulted in increased mitochondrial density, biogenesis (PGC1α and PPARγ), and increased markers of a beige adipocyte phenotype (UCP1, TBX1, and β-3AR). This upregulation in mitochondrial expression was accompanied by increases oxygen consumption. In GS-treated macrophages, markers of M2 polarization were elevated (e.g., arginase and IL-10), along with increased catecholamine release into the media. Lastly, 3T3-L1 adipocytes treated with conditioned media from macrophages induced a 167.8% increase in UCP1 expression, suggestive of a role of macrophages in eliciting an anti-adipogenic response to GS. **Conclusions:** Results from this study provide the first mechanistic understanding of the anti-obesity effects of GS and suggests a role for both direct GS-signaling and indirect stimulation of M2 macrophage polarization in this model.

## 1. Introduction

Obesity is a complex disease associated with a pathological expansion of white adipose tissue. Adipose tissue has been traditionally classified into the energy-storing white adipose tissue and energy-dissipating, thermogenic brown adipose tissue. In specific environmental conditions, such as elevated beta-adrenergic signaling, white adipose tissue can upregulate mitochondrial biogenesis, uncoupling protein-1 (UCP1) expression, and respiration [1]. This change in the metabolic potential has been termed “beiging” both because of the browning in color of adipocytes in addition to a phenotype that is more metabolically active than white adipose but not to the extent of brown adipose tissue. Increases in beiging have been demonstrated in rodents exposed to cold [2] and chemical agents [3], which can increase metabolic rate, dispense excess energy, and promote weight loss (or protection from weight gain). Due to the expansiveness of the white adipose depot over brown depots in obesity, understanding how the process of beiging works and how it can be manipulated is a rapidly evolving area of research.

Many plant-derived compounds have anti-obesity effects, and are reported to prevent adipogenesis and promote lipolysis in both in vitro and in vivo models [4,5]. The specific mechanisms that drive these responses, however, are not as well described. It has been hypothesized that many phytochemicals may have the potential to induce transdifferentiation of adipocytes to a beige phenotype [6]. For example, resveratrol is a potent activator of the sirtuins [7], which independently upregulates peroxisome proliferator-activated receptor gamma coactivator 1-alpha (PGC1α) and mitochondrial biogenesis [8]. Further, novel work has demonstrated that phytochemicals may stimulate macrophage M2 polarization, which promotes the release of both anti-inflammatory cytokines [9] and as hypothesized in the current study, catecholamines. Interestingly, recent work has evidenced that macrophage M2 polarization may contribute to adipocyte beiging and energy expenditure [10]. Pro-inflammatory macrophage infiltration into the adipose depot is a characteristic of obesity [11,12]. Thus, conversion of resident macrophages to catecholamine-secreting cells may promote localized beta-adrenergic signaling and induce beiging in adipose tissue [13,14]. Despite such intriguing links, little work to date has explored the beiging potential of phytochemicals in either direct or indirect methods of adipocyte beiging.

Guggulsterone (GS) is a bioactive phytochemical derived from the guggul plant. It is both an agonist and antagonist for various steroid receptors and thus, has been suspected to have beneficial effects in cancer [15] and cardiovascular disease prevention [16], and as an anti-obesity compound. Previous work from our lab has shown that GS treatment in 3T3-L1 adipocytes can impair differentiation and induce both lipolysis and apoptosis in mature adipocytes [17]. Further, these beneficial effects on adipogenesis can be heightened with the addition of genistein in the media [18]. In vivo, GS promotes a beneficial lipid profile in rats fed a high fat diet, along with an improvement in glucose control [19]. Further, it has also been shown that GS can blunt weight gain in rats by reducing food intake, suggesting the anti-obesity properties may be multifaceted [20]. GS is an agonist for the bile acid receptors, which has been identified as a potential regulator of thyroid hormone signaling [21]. As elevations of triiodothyronine (T3) induce an upregulation of UCP1, it may be plausible that GS induces adipocyte beiging through modulating T3 synthesis. Further, GS has anti-inflammatory properties in cultured macrophages [22]. Hence, GS may also function as a beige adipogenic factor through indirect mechanisms involving M2-polarization. To our knowledge, the efficacy of GS to stimulate beiging of adipocytes has yet to be investigated.

A recent set of studies demonstrated that 3T3-L1 adipocytes are a useful model of beiging [23]. In mature 3T3-L1 adipocytes, beta-adrenergic stimulation induces UCP1 upregulation and increases cellular oxygen consumption by reducing coupling efficiency [24]. Utilizing this model, the current study sought to determine if the anti-obesity phytochemical, GS, can promote mitochondrial biogenesis and beiging in 3T3-L1 adipocytes. Furthermore, we investigated if this could be mediated through direct stimulation of adipocytes and by indirect stimulation through macrophage M2 polarization and catecholamine release. Findings from this study provide evidence that GS-treatment induces adipocyte beiging through both direct stimulation of mitochondrial biogenesis and indirectly by the polarization of macrophages that can signal to adipocytes and activate adrenergic pathways.

## 2. Materials and Methods 

### 2.1. Cell Culture

Mouse embryo 3T3-LI preadipocytes were purchased from Zenbio (Research Triangle Park, NC, USA) and RAW264.7 macrophages were purchased from America Type Culture Collection (Manassas, VA, USA). 3T3-L1 cells were cultured in Dulbecco’s Modified Eagle Medium (DMEM/F12) GlutaMAX with 10% calf serum (CS; Thermo Fisher Scientific, Waltham, MA, USA), and 1% penicillin streptomycin (PS; Sigma-Aldrich, St. Louis, MO, USA). 3T3-L1 cells were grown to confluence and induced to differentiate using differentiation media I (DM I) containing DMEM/F12 GlutaMAX with 10% fetal bovine serum (FBS; Thermo Fisher Scientific, Waltham, MA, USA), 1% PS, 5 μM dexamethasone (Dexa), 1 mg/mL insulin, 0.5 mM 3-isobutyl-1-methylxanthine, and 1 μM rosiglitazone (Sigma-Aldrich, St. Louis, MO, USA). Cells were maintained in DM I for 3 days followed by another 4–6 days in DM II which contained DMEM/F12 GlutaMAX with 10% FBS, 1% PS, 1 mg/mL insulin, and 1 µM Dexa. Media was changed every other day during the process of differentiation and by day 8, 90–95% of cells were mature adipocytes with lipid droplets. Adipocyte-specific assays are described in the following sections.

The murine monocytic cell line, RAW264.7, was cultured in Roswell Park Memorial Institute media (RPMI; ThermoFisher Scientific, Waltham, MA, USA) complete with 10% FBS and 1% PS for macrophage polarization experiments. After 95% confluency, RAW264.7 cells were treated with 0.1% DMSO vehicle, (E)-guggulsterone (GS; Sigma-Aldrich, St. Louis, MO, USA) 6 µM or IL-4 20 ng/mL (interleukin-4; R&D Systems, Minneapolis, MN, USA).

For conditioned media experiments, RAW264.7 cells were cultured in DMEM/F12 GlutaMAX supplemented with 10% FBS and 1% PS. After 48 h of treatment with GS (6 µM) or IL-4 (20 ng/mL), conditioned media was collected from RAW264.7 cells and transferred to mature 3T3-L1 adipocytes for 24 h.

### 2.2. Lipid Quantification

Lipid droplets were stained in mature adipocytes treated with varying doses of GS (6–25 µM) or 0.1% DMSO vehicle control during the differentiation period using the AdipoRed™ reagent (Lonza Inc., Walkersville, MD, USA) according to manufacturer’s protocol. The microplate was read using the HT Synergy (BioTek, Winooski, VT, USA) microplate reader.

### 2.3. Cell Viability

Mature adipocytes and RAW264.7 macrophages were treated with GS or 0.1% DMSO vehicle control for 24 and 48-h, respectively. Cell viability was measured after treatment using Prestoblue® Cell Viability Reagent (Thermo Fisher Scientific, Waltham, MA, USA) according to manufacturer’s protocol. Absorbance of metabolically active cells was measured one hour after incubation using an HT Synergy microplate reader at 570 nm.

### 2.4. Mitochondrial Biogenesis

Mature 3T3-L1 adipocytes were treated with GS (25 µM), isoproterenol (10 µM), or 0.1% DMSO vehicle control for 24 h and incubated with MitoTracker® Green FM (Thermo Fisher Scientific, Waltham, MA, USA) according to manufacturer’s instructions. Mitochondrial activity was measured using the HT Synergy microplate reader.

### 2.5. Oxygen Consumption

Mature adipocytes were treated with GS (25 µM) and isoproterenol (10 µM), or 0.1% DMSO vehicle for 24 h. The Oxygen Consumption/Glycolysis Dual Assay Kit (Cayman Chemical, Ann Arbor, MI, USA) was used to detect oxygen consumption following manufacturer’s protocols. Fluorescence of the MitoXpress® Xtra reagent was measured using the HT Synergy microplate reader.

### 2.6. Immunoblot Analysis

Following treatment with GS for 24 or 48 h, cells were suspended with ice cold RIPA Lysis and Extraction buffer, complete with protease and phosphatase inhibitors (Thermo Fisher Scientific, Grand Island, NY, USA). Whole cell lysate was prepared by centrifuging cells for 10 minutes at 13,300× *g*. Protein estimation was then determined using the Pierce BCA Protein Assay Kit (Thermo Fisher Scientific, Grand Island, NY, USA). Proteins were separated on discontinuous SDS 4–20% polyacrylamide gels and transferred onto a polyvinylidene difluoride (PVDF) membrane using a Trans-blot Turbo system (Bio-Rad, Hercules, CA, USA) and then blocked for one hour with Tris buffered saline plus 0.1% Tween 20 (TBS-T) containing 5% bovine serum albumin (BSA). Following blocking, blots were then incubated for one hour in TBS-T plus 5% BSA with primary antibodies. Blots were incubated with secondary antibodies conjugated to IRDye 800 and developed using the Odyssey CLX imaging system (LiCor Biosciences, Lincoln, NE, USA). Signal intensity was determined for each band using the LiCOR imaging system software (Image Studio Ver. 5.2, LiCor Biosciences, Lincoln, NE, USA). For each protein of interest, the density value was normalized to the corresponding density of the loading control to obtain the integrated density values. These values were then normalized to control samples run on the same gel.

Primary antibodies used were: anti-β-Actin (sc-47778; 1/10,000 dilution) from Santa Cruz Biotechnology Inc. (Dallas, TX, USA). Anti-GAPDH (glyceraldehyde 3-phosphate dehydrogenase; ab9484; 1/10,000 dilution), anti-UCP1 (ab10983; 1/2000 dilution), anti-liver-arginase-1 (ab124917; 1/2000 dilution), and anti-TBX1 (t-box transcription factor 1; ab18530; 1/2000 dilution) antibodies were from Abcam (Cambridge, United Kingdom). Anti-β-3AR (β-3 adrenergic receptor; PA5-50914; 1/500 dilution) was from Thermo Fisher Scientific (Grand Island, NY, USA). Lastly, anti-α-Tubulin (NB100-690; 1/10,000 dilution), anti-PPARγ (peroxisome proliferator-activated receptor-γ; NBP2-22106; 1/1,000 dilution), and anti-PGC-1α (peroxisome proliferator-activated receptor-γ coactivator 1-α; NBP1-04676SS; 1/1000 dilution) antibodies were purchased from Novus Biologicals (Littleton, CO, USA). Membranes were incubated in their respective primary antibody for 1 h at room temperature.

### 2.7. Catecholamine Assay

Following 48-h incubation with GS in RAW264.7 cells, the culture supernatant was removed to quantify catecholamine release. Catecholamine levels were determined using ELISA according to manufacturer’s instructions (Blue Gene for Life Science, Shanghai, China). The assay plate was read on a HT Synergy microplate reader.

### 2.8. Interleukin-10 (IL-10) ELISA

Following 24-h incubation with GS, the culture supernatant was removed to quantify IL-10 secretion. IL-10 levels were determined using ELISA according to manufacturer’s instructions (R&D Systems, Minneapolis, MN, USA). The assay plate was read on a HT Synergy microplate reader at 450 nm.

### 2.9. Data Analysis

All data was normalized to control and expressed as the mean ± S.E.M. Either t-tests or Dunnett’s multiple comparisons test was used to determine difference between treatment groups and control where appropriate. Statistical analysis and graphs were made using GraphPad Prism (version 6.07; La Jolla, CA, USA). Statistically significant differences are defined at *p* < 0.05.

## 3. Results

### 3.1. GS inhibits Adipogenesis in 3T3-L1 Adipocytes 

**Figure 1:**
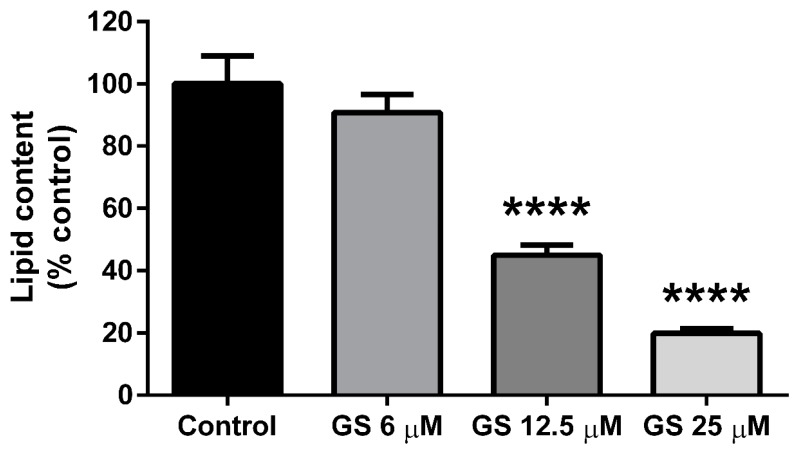
Guggulsterone (GS) reduced lipid accumulation in 3T3-L1 adipocytes. GS treatment during the differentiation period in 3T3-L1 adipocytes reduces adipogenesis in a dose dependent manner. Data presented as mean ± SEM from *n* = 3–6 replicates per group. **** *p* < 0.0001 vs. control.

3T3-L1 preadipocytes were cultured with GS (6, 12.5, 25 µM) during the adipogenesis period for 6 days. Both the 12.5 and 25 µM dose of GS reduced total lipid content in adipocytes following treatment (55.1 ± 11.9%, *p* < 0.0001 and 80.2 ± 8.9%, *p* < 0.0001 respectively; Figure 1). The lowest dose of GS (6 µM) did not significantly reduce lipid accumulation in mature 3T3-L1 adipocytes. These results are in agreement with our previous findings on the anti-adipogenic effects of GS [16,17].

### 3.2. Effect of GS on 3T3-L1 Viability and Apoptosis

**Figure 2:**
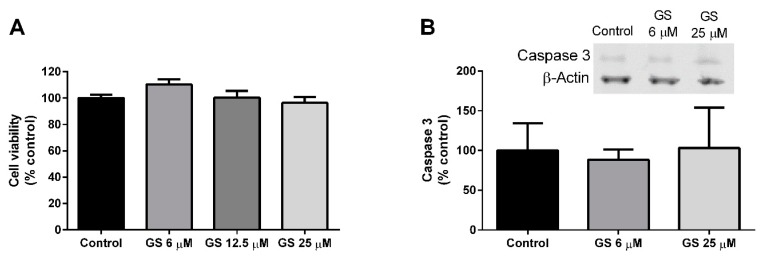
GS treatment does not impact viability of 3T3-L1 adipocytes. GS treatment for 24 h in mature, 3T3-L1 adipocytes has no effect on cell viability (**A**) or apoptosis as measured by caspase 3 (**B**). Data presented as mean ± SEM from *n* = 3–6 replicates per group.

Mature 3T3-L1 adipocytes were cultured with GS (6, 12.5, 25 µM) for 24-h. Following treatment, there was no impact of GS on cell viability (Figure 2A) or caspase-3 activation (Figure 2B), indicating that GS does not produce cytotoxic effects in adipocytes.

### 3.3. Effect of GS on Mitochondrial Biogenesis and Oxygen Consumption

**Figure 3:**
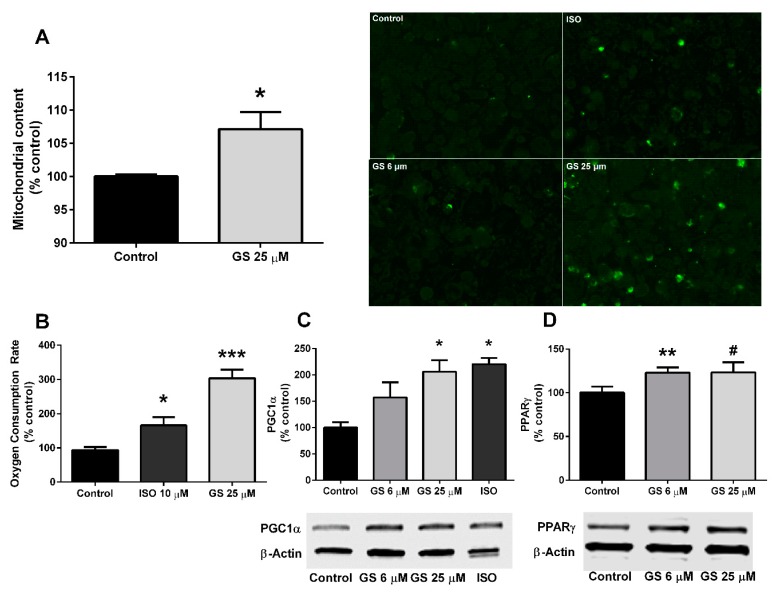
GS treatment in 3T3-L1 adipocytes induces mitochondrial biogenesis. GS treatment increases mitochondrial density (20× objective) (**A**), oxygen consumption rate (**B**), and markers of mitochondrial biogenesis (**C** and **D**). Data presented as mean ± SEM from *n* = 3–6 replicates per group. # *p* < 0.10, * *p* < 0.05, ** *p* < 0.01, *** *p* < 0.001 vs. control. Abbreviations: isoproterenol (ISO), peroxisome proliferator-activated receptor gamma coactivator 1-alpha (PGC1α), peroxisome proliferator-activated receptor gamma (PPARγ).

Mitochondria were quantified following a 24-h treatment with GS through fluorescence intensity of Mitotracker staining. Mitochondrial density increased by 7.2 ± 2.3% (*p* < 0.05) at the 25 µM dose (Figure 3A), which resulted in a significant upregulation of oxygen consumption compared to untreated cells (210 ± 23.9%, *p* < 0.001; Figure 3B). GS at 25 µM also increased the concentration of PGC1α (105.7 ± 22.2%, *p* < 0.05; Figure 3C) compared to control, DMSO-treated cells, and a small increase in PPARγ was observed, but did not reach significance (*p* = 0.07; Figure 3D). GS at the 6 µM concentration failed to increase PGC1α, however the dose increased PPARγ compared to control (22.9 ± 6.1%; *p* < 0.01).

### 3.4. GS-Induced Effects on Markers of Beige Adipocytes

**Figure 4:**
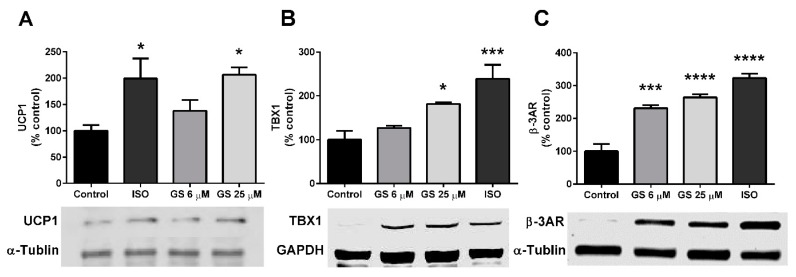
GS treatment increases markers of beiging in 3T3-L1 adipocytes. GS treatment upregulates markers of beiging, including UCP1 (**A**), TBX1 (**B**), and β-3AR (**C**) proteins. Data presented as mean ± SEM from *n* = 4 replicates per group. * *p* < 0.05, *** *p* < 0.001 vs. control. Abbreviations: isoproterenol (ISO), uncoupling protein 1 (UCP1), glyceraldehyde 3-phosphate dehydrogenase (GAPDH), T-box protein 1 (TBX1), β-3 adrenergic receptor (β-3AR).

Markers of beiging, notably the presence of UCP1, TBX1, and β-3AR were quantified in GS-treated cells. 25 µM GS increased UCP1 (106.8 ± 23.8%, *p* < 0.05; Figure 4A) and TBX1 (81.6 ± 3.3%, *p* < 0.05; Figure 4B) compared to the control. β-3AR was upregulated with treatment of both 6 µM and 25 µM (131.2 ± 24.7% and 164.3 ± 21.4%, respectively; Figure 4C) compared to control-treated cells. This increase in markers of beiging was to a similar extent to what was observed when mature adipocytes were cultured with the beta agonist, isoproterenol for 24 h.

### 3.5. Effect of GS on M2 Polarization of Macrophages

To determine if GS indirectly promotes beiging in adipocytes through macrophage M2 polarization, viability was first determined in GS-treated RAW264.7 macrophages following 24 h of treatment (data not shown). GS impaired viability at 12.5 µM (18.0 ± 3.0%, *p* < 0.001) and 25 µM (25.6 ± 3.4%, *p* < 0.0001), therefore, GS 6 µM was chosen as an optimal concentration.

**Figure 5:**
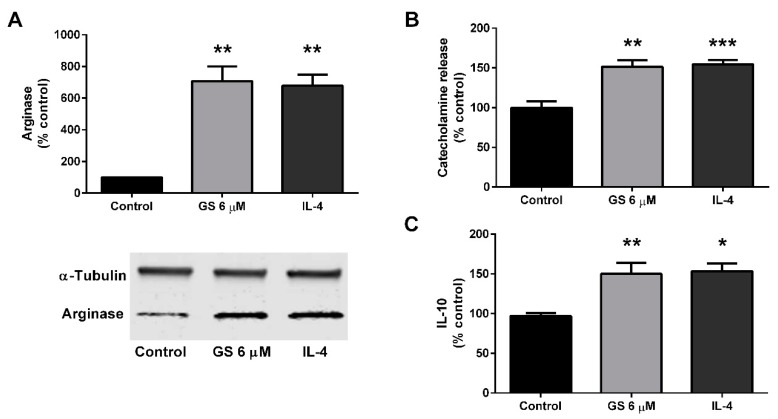
GS treatment in RAW264.7 macrophages induces M2 polarization. GS treatment induces an M2-like phenotype in macrophages as suggested by arginase activation (**A**), catecholamine release into the media (**B**), and release of IL-10 (**C**). Data presented as mean ± SEM from *n* = 3–6 replicates per group. * *p* < 0.05, ** *p* < 0.01, *** *p* < 0.001. Abbreviations: interleukin 4 (IL-4).

The M2 polarization of the RAW264.7 macrophage cell line was then assessed following GS treatment. RAW264.7 macrophages were then cultured with 6 µM GS or IL-4 for 48 h to determine if treatment resulted in an M2-like phenotype. Both IL-4 control (578.3 ± 68.5%, *p* < 0.01) and GS treatment (608 ± 91.7%; *p* < 0.01) upregulated arginase expression (Figure 5A). Along with arginase upregulation, treatment sufficiently stimulated catecholamine release at approximately the same level for both IL-4 control (54.6 ± 5.4%, *p* < 0.001) and 6 µM GS (51.5 ± 8.0%, *p* < 0.01; Figure 5B). Similarly, IL-10 release in the media was also upregulated with 6 µM GS treatment compared to control (49.9 ± 9.8%, *p* <0 .01; Figure 5C). Together, these data suggested that GS induced M2 activation of macrophages at the same extent to that of IL-4 treatment.

### 3.6. Role of M2 Macrophage Polarization on GS-Induced on Adipocyte Beiging

**Figure 6:**
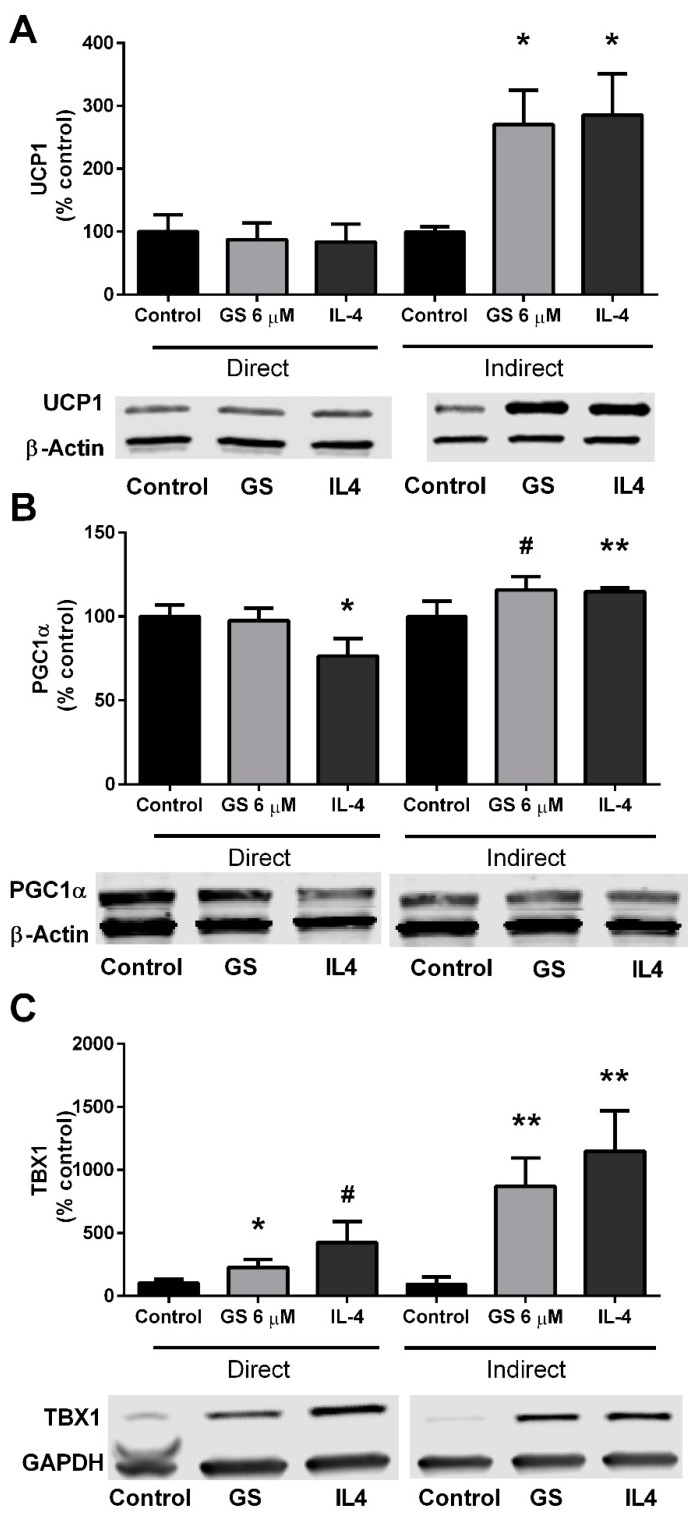
Conditioned media from GS-treated macrophages induces UCP1 upregulation in adipocyte. GS and IL-4 treatment upregulate UCP1 (**A**), PGC1α (**B**), and TBX1 (**C**) expression through direct stimulations in 3T3-L1 adipocytes and indirectly via conditioned media from RAW264.7-treated macrophages. Data presented as mean ± SEM from *n* = 3–4 replicates per group. # *p* < 0.10, * p < 0.05, ** *p* < 0.01 vs. control. Abbreviations: Uncoupling protein 1 (UCP1), interleukin 4 (IL-4), peroxisome proliferator-activated receptor gamma coactivator 1-alpha (PGC1α), T-box protein 1 (TBX1).

Lastly, in an additional experiment, conditioned media from GS-treated RAW264.7 macrophages were plated onto mature 3T3-L1 adipocytes. UCP1 was upregulated in the adipocytes (170.7 ± 54.1%; *p* < 0.01) compared to untreated RAW264.7 conditioned media (Figure 6A). This upregulation was to a similar extent of IL-4-stimulation (185.5 ± 65.8%). PGC1α was increased by 15.8 ± 8.0% (*p* = 0.06) by indirect GS treatment and 14.8 ± 2.1% (*p* < 0.01) by indirect IL-4 treatment compared to un-treated conditioned media (Figure 6B). Lastly, a marker of beige adipocytes was also analyzed following 24 h of conditioned media treatment; TBX1 was increased with both GS (771.0 ± 224.7%; *p* < 0.01) and IL-4 (1047 ± 322.1%; *p* < 0.05) conditioned media (Figure 6C).

## 4. Discussion

The current study expands on the mechanistic understanding of the anti-obesity effects of GS in adipocytes in vitro. Mature 3T3-L1 adipocytes treated with GS demonstrated increased markers of mitochondrial biogenesis and UCP1 expression, indicative of an induction of a beiging phenotype. As adipose tissue contains macrophages, in addition to adipocytes, this study also explored the effects of GS on RAW264.7 cells. Macrophages treated with GS increased arginase expression, stimulated the secretion of IL-10, an inflammatory cytokine, and upregulated catecholamine release into the media indicative of M2 polarization. Further, conditioned media from GS-treated macrophages were sufficient enough to induce UCP1 expression in adipocytes.

GS has been proposed to be an anti-obesity phytochemical. In vitro treatment of GS results in reduced lipid accumulation, partly by inducing apoptotic pathways [25] and reducing adipogenic signaling in differentiating adipocytes [16,17]. Herein, we demonstrate that a novel anti-obesity property of GS is the induction of beiging. GS shares a structural similarity to many of the steroid hormones and has both agonistic and antagonistic capabilities on a number of steroid receptors including the Takeda G-protein-coupled receptor 5 (TGR5) bile acid receptor, estrogen receptor, and the progesterone receptor. Hence, it is likely that the reason that GS has been suggested to target diseases ranging from cancer to cardiometabolic health is attributable to its ability to interact with a broad spectrum of steroid receptors. Sex hormones have been hypothesized to influence the thermogenic potential of brown adipose tissue, with both estrogen and progesterone having positive effects on UCP1 expression [26]. Unsurprisingly, activation of estrogen receptor alpha in 3T3-L1 adipocytes induces expression of UCP1 and TBX1 through phosphorylation of AMP-activated protein kinase (AMPK) [27], which is a critical sensor of energy balance and an upstream regulator of UCP1 [28]. Further, activation of the TGR5 bile acid receptor can independently induce thermogenesis in brown adipose tissue [21,29]. TGR5 signaling upregulates DIO2 expression, which may increase the UCP1 expression necessary for thermogenesis. Together, this suggests that GS may induce beiging in adipocytes through several potential signaling cascades, which could have been responsible for the upregulation of UCP1 and oxygen consumption observed in the current study. However, this also makes it difficult to understand the specific signaling mechanisms by which GS may be working through in adipocytes.

While we are unable to explain how GS directly induces a beige phenotype in the 3T3-L1 adipocyte, we sought to explore if signals from other peripheral cell types in adipose tissue may influence this phenomenon. Specifically, macrophages can be found in concentrations of upwards of 40% of the cell types in adipose tissue from obese subjects [11] and thus, makes macrophages an important target for anti-obesity compounds. A small subset of studies exist that investigate the effects of GS on macrophages. Perhaps most relevant, a recent study by Che et al. [30] reported that treatment of macrophages with GS in vitro increased markers of M2 polarization in RAW264.7 cells, primary mouse macrophages, and human monocytes. M2 polarization of colonic macrophages was also found in vivo. A shift away from the M1 phenotype in RAW264.7 cells have also been reported in other work [22,31], and together, is supportive of the findings in the current study and the potential for GS to induce beiging through stimulation of resident macrophages.

M2 macrophage polarization occurs during the Th2 response, is activated by IL-4 and glucocorticoids, and release cytokines such as transforming growth factor-β (TGF-β) which can orchestrate the tissue remodeling process. M2 polarization, purportedly as a result of IL-4 stimulation, may also increase norepinephrine production and release [32]. It is hypothesized that cold-induced sympathetic nervous system stimulation induces tyrosine hydroxylase enzyme activity in macrophages, thereby increasing norepinephrine release capable of white adipose tissue beiging. This response has been demonstrated in several rodent models [12], however additional work has recently questioned the relevance of alternatively activated macrophages in adipose tissue [33]. In Fischer et al., macrophage-specific knockout of tyrosine hydroxylase had no impact on body temperature during a cold challenge [33]. Further, in vitro IL-4 stimulation of bone marrow-derived macrophages also failed to induce an adipocyte thermogenic profile in conditioned media experiments, and in vivo IL-4 treatment failed to increase energy expenditure in mice. This is in conflict with the findings in the current study, however, the discrepancies may be related to differences in methodology.

The current study utilized the 3T3-L1 adipocyte line with a differentiation media that contained thyroid hormone, which is important for producing an adipocyte sensitive to UCP1 upregulation [23]. Unlike the aforementioned study by Fischer et al. (2017), which only cultured for 24 h [33], adipocytes in the current study were cultured in the conditioned media for 48 h. The additional time in a primed cell may partially explain our results. Further, it is plausible that continued direct GS signaling from the macrophage-media source may have contributed to the observed UCP1 upregulation. Thus, it is reasonable to hypothesize that it may be necessary to have both systemic and resident macrophage-directed adrenergic signaling to stimulate adipocytes. Lastly, the studies in Fischer et al., failed to see changes in metabolic outputs in mice with deficiencies in either tyrosine hydroxylase or IL-4 [33]. In fact, it may be that alternatively activated macrophages contribute, but are not a primary factor to beiging in vivo. Obesity-related macrophage infiltration in the adipose tissue may further add to this contribution, as what was recently shown in cultured adipocytes treated with conditioned media from primary macrophages obtained from obese donors [34].

The pharmacologic translation of in vitro studies to in vivo remains a challenge in assessing the efficacy of phytochemicals to act as attenuators of disease. In the current study, we used the trans (E)-form of GS for dosing, which we have previously demonstrated has less lipolytic potential than the cis (Z)-form [17]. However, the cis (Z)-form is more cytotoxic [17], which attributed to the selection of the trans (E)-form herein as we wanted to avoid the confounding factor of cell death from influencing the results. It remains plausible that these two isoforms may have differing potentials to beige in vitro, and considering the rapid metabolism of GS in vivo, may also vary widely in a whole-body system. The Cmax for GS is 0.3 µM in rats [35], hence an important limitation of the current study is the supraphysiological doses used. Nonetheless, GS has demonstrated synergistic properties with other phytochemcials such as genistein [18], which would permit for the plausibility of a photochemical blend that contained more physiologic relevant concentrations. Furthermore, hypolipidemic and anti-diabetic effects of GS were demonstrated in humans [36] and rodents [19] suggesting potential beneficial effects of GS under in vivo conditions.

## 5. Conclusions

**Figure 7:**
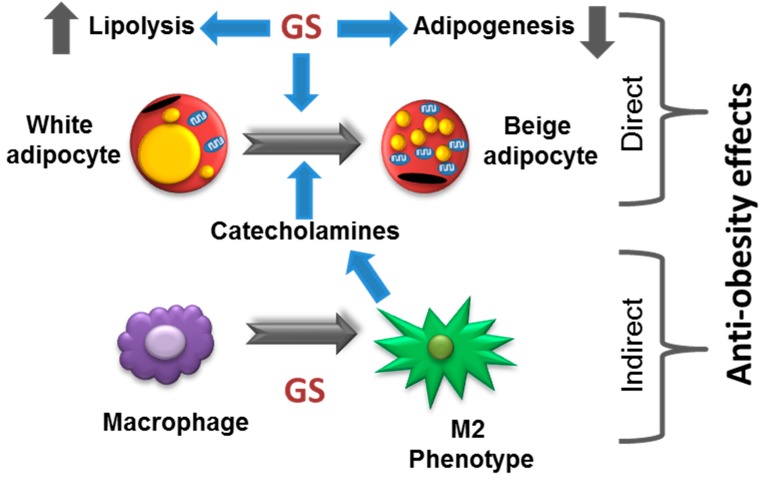
Proposed model of anti-obesity effects of GS.

Herein, findings from this work demonstrate that the anti-obesity effects of GS include beiging of adipocytes in vitro. GS has direct activity on 3T3-L1 adipocytes, inducing mitochondrial biogenesis and an upregulation of UCP1 and cellular oxygen consumption, indicative of a beige phenotype. Further, GS appears to have the capability of promoting adipocyte beiging through a macrophage-dependent mechanism. In the current study, treatment with GS in RAW264.7 macrophages promoted M2 polarization and subsequent catecholamine release that was capable of upregulating UCP1 in 3T3-L1 adipocytes. While preliminary results indicate that GS-induced enhancement of beiging may be due to both direct and indirect signaling in cultured adipocytes (summarized in Figure 7). Further studies are required to demonstrate this phenomenon in vivo.
